# Dynamical predictive coding with reservoir computing performs noise-robust multi-sensory speech recognition

**DOI:** 10.3389/fncom.2024.1464603

**Published:** 2024-09-23

**Authors:** Yoshihiro Yonemura, Yuichi Katori

**Affiliations:** ^1^Graduate of System Information Science, Future University Hakodate, Hakodate, Hokkaido, Japan; ^2^International Research Center for Neurointelligence (IRCN), The University of Tokyo, Tokyo, Japan

**Keywords:** multi-sensory integration, predictive coding, reservoir computing, speech recognition, nonlinear dynamics

## Abstract

Multi-sensory integration is a perceptual process through which the brain synthesizes a unified perception by integrating inputs from multiple sensory modalities. A key issue is understanding how the brain performs multi-sensory integrations using a common neural basis in the cortex. A cortical model based on reservoir computing has been proposed to elucidate the role of recurrent connectivity among cortical neurons in this process. Reservoir computing is well-suited for time series processing, such as speech recognition. This inquiry focuses on extending a reservoir computing-based cortical model to encompass multi-sensory integration within the cortex. This research introduces a dynamical model of multi-sensory speech recognition, leveraging predictive coding combined with reservoir computing. Predictive coding offers a framework for the hierarchical structure of the cortex. The model integrates reliability weighting, derived from the computational theory of multi-sensory integration, to adapt to multi-sensory time series processing. The model addresses a multi-sensory speech recognition task, necessitating the management of complex time series. We observed that the reservoir effectively recognizes speech by extracting time-contextual information and weighting sensory inputs according to sensory noise. These findings indicate that the dynamic properties of recurrent networks are applicable to multi-sensory time series processing, positioning reservoir computing as a suitable model for multi-sensory integration.

## 1 Introduction

Multi-sensory integration is a fundamental process through which the brain combines information from different sensory modalities, such as sight, sound, touch, smell, and taste, to form a comprehensive understanding of the environment (Stein and Stanford, 2008). This integration allows for more accurate and reliable perception than would be possible through any single sensory modality alone. The primary purpose of multi-sensory integration is to enhance the detection, localization, and identification of stimuli in the environment. For example, seeing a speaker's lips move in sync with the sounds they produce helps in understanding speech, especially in noisy environments (McGurk and MacDonald, 1976; Radeau and Bertelson, 1977). Similarly, combining the tactile and visual aspects of an object can provide a more detailed perception of its properties, like texture and shape (Botvinick and Cohen, 1998). Understanding multi-sensory integration has significant implications across various fields. In education, understanding multi-sensory integration helps develop teaching methods that use multiple senses to make learning more effective. In technology, specifically in autonomous vehicles and robotics, this multi-sensory integration is essential for processing complex environmental data through multiple sensors, ensuring safer navigation. Furthermore, in healthcare, multi-sensory therapeutic approaches aid in rehabilitation, such as in stroke recovery, and the development of sensory-feedback prosthetics, significantly improving patient outcomes. Multi-sensory integration research is being conducted from multiple perspectives using psychological, neurological, and computational approaches.

In a psychological experiment on perception, it was demonstrated that the integration of auditory and visual cues significantly enhances speech recognition. This improvement is particularly notable in noisy environments (Stevenson and James, 2009). The principle of “inverse effectiveness” asserts that combining sensory cues from different modalities is more advantageous when the effectiveness of those individual cues is reduced. In essence, when single sensory signals are weak or obscured by factors such as environmental noise or poor visibility, their combined integration leads to significantly enhanced perceptual accuracy and response efficiency. This is especially critical in situations where a single sensory modality is insufficient for accurate perception. For instance, in noisy situations where auditory information alone might fail to convey a message clearly, the addition of visual cues, such as lip movements, can significantly improve speech comprehension. Inverse effectiveness highlights the adaptive advantage of multi-sensory integration, enabling organisms to maintain high perceptual and behavioral performance in challenging conditions. Despite the clear benefits of multi-sensory integration as outlined by the principle of inverse effectiveness, the detailed mechanisms underlying this process remain largely unexplored.

In neurological studies, multi-sensory integration is believed to occur in several brain regions, with the superior colliculus in the midbrain being a well-documented site for visual-auditory integration (Stein and Stanford, 2008). Other areas, such as the cortex, have regions specialized for integrating specific types of sensory information (Ghazanfar and Schroeder, 2006). Neural processes involved in multi-sensory integration can enhance the brain's representation of objects, leading to faster reaction times and improved accuracy in response to stimuli (Calvert and Thesen, 2004).

The process of multi-sensory integration is thought to involve the summation of multiple sensory modalities weighted by their reliability. A key piece of neurological evidence is the functional connectivity among cortical regions related to visuo-tactile integration (Beauchamp et al., 2010). This study illustrated that functional connectivity between higher integration areas and lower sensory areas diminishes when the respective sensory information is considered unreliable. Such variability in functional connectivity among brain areas has also been confirmed through experiments on speech recognition tasks (Nath and Beauchamp, 2011). Additionally, physiological experiments have implied a common mechanism, evidenced by the activation in diverse brain regions when participants perform different tasks involving the same combination of modalities (Stevenson and James, 2009).

The computational theory of multi-sensory integration, significantly enhanced by Bayesian causal inference, provides a probabilistic framework that elucidates how the brain synthesizes information from various senses. This theory posits that the brain employs a Bayesian approach to assess the likelihood that different sensory inputs originate from a common source, thereby optimizing perceptual accuracy (Knill and Pouget, 2004). Incorporating Bayesian principles has deepened our understanding of the inferential processes underlying unified sensory experience from disparate inputs, treating sensory integration as a dynamic cognitive process rather than a straightforward mechanical merger (Ernst and Banks, 2002; Alais and Burr, 2004). This advancement not only bridges computational neuroscience and cognitive psychology but also paves the way for AI systems with enhanced sensory processing capabilities (Doya, 2007). Furthermore, based on the Bayesian causal inference model, brain regions involved in estimating stimulus positions from audio-visual information were explored using fMRI (Rohe and Noppeney, 2015). This measurement revealed that non-integrated sensory information activity occurred in lower sensory areas, while integrated sensory information activity was observed in higher areas, suggesting hierarchical multi-sensory integration. These discoveries suggest the presence of a reliability-weighting mechanism rooted in the Bayesian causal inference model within the brain. Although physiological evidence supports the computational mechanism for reliability-weighted integration, the precise neural substrates responsible remain to be fully elucidated.

Another promising computational approach for the perception mechanism is the predictive coding theory, positing that the brain continuously generates and updates predictions about sensory inputs (Rao and Ballard, 1999). This perspective deepens our understanding of the neural processes behind sensory information processing and highlights the dynamic nature of perception, where predictive models are constantly refined through interaction with the external world. Predictive coding attributes specific functions to bidirectional streams: the higher area predicts the states of the lower area with top-down signaling, while the lower area sends prediction errors to the higher area with bottom-up signaling. The internal state of each area is updated to minimize the prediction error by the bidirectional information exchange. In a hierarchical network model of predictive coding, the internal network, which is referred to as the generative model, predicts the sensory signal and the prediction error is utilized to refine the state of the internal network. The predictive coding theory has been widely studied not only in the primary sensory area but also in the higher brain areas such as the prefrontal cortex (Kilner et al., 2007; Alexander and Brown, 2018).

Attempts to elucidate multi-sensory integration within the predictive coding framework suggest that signal reliability, meaning the confidence in the accuracy of sensory information, significantly shape perception (Talsma, 2015). In multi-sensory integration, the reliability of a signal refers to the confidence in the accuracy of sensory information. More reliable signals have less variance and are more likely to influence perception. Error feedback is crucial in updating internal models based on the mismatch between expected and actual sensory inputs. Attention modulates this process by prioritizing certain stimuli, enhancing the integration of relevant information, and suppressing irrelevant data. This interplay ensures that our perception is both accurate and adaptable to changes in our environment. The neural network model of the multi-sensory integration based on the predictive coding has also been proposed (Spratling, 2016). This model illustrates the process of integrating multiple sensory information in the spatial localization task. This model describes the dynamics involved in multi-modal information processing of perception, but it is not yet sufficient to handle complex time-varying sensory signals, such as speech or fluctuating visual signals.

In our previous study, we constructed a multi-sensory integration model based on predictive coding with reservoir computing that can reconstruct visual information from auditory information by associating different sensory modalities (Yonemura and Katori, 2021). This model can process complex time series, such as vocal patterns, by utilizing a large recurrent network as the generative model of predictive coding. Here, we employed the idea of reservoir computing, which is one of the recurrent neural network models that has the advantage of processing dynamically changing time sequences (Jaeger, 2002). In this paper, we propose the multi-sensory integration model based on predictive coding with reservoir computing. We extend the multi-sensory integration model by incorporating a reliability-weighting mechanism. In the following sections, we describe a reservoir-based predictive coding model that models the hierarchical structure of the cortical network. The model is then evaluated using a multi-modal speech recognition task and show that reliability weighting plays an important role in the task.

## 2 Material and methods

In the process of multi-sensory integration, multiple sensory areas and the cortical areas that integrate these sensory inputs work together. The cortex has common neural structures: local recurrent connectivity and sparse connectivity among different areas. To develop the model of the multi-sensory integration, we modeled the local connectivity based on reservoir computing and modeled the connectivity among different areas by the hierarchical network structure based on predictive coding.

**Figure 1:**
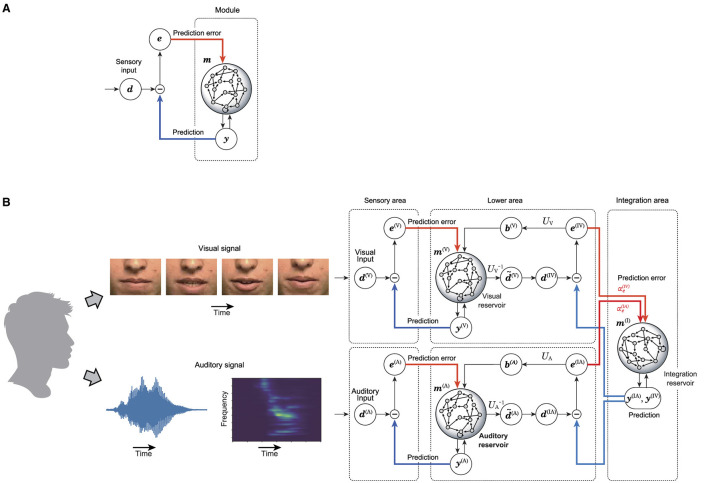
Proposed model in this research. **(A)** Shows the predictive coding with reservoir computing model (PCRC) which composes the multi-sensory integration model. **(B)** Shows the whole architecture of the multi-sensory integration model.

Each area consists of the reservoir-based predictive coding model, which is depicted in Figure 1A. This module includes a layer of sensory inputs, a prediction layer, a recurrent network acting as a reservoir, and a prediction error layer. The learning process involves adjusting the weights from the reservoir to the prediction layer to enable accurate reconstruction of the provided sensory signal. The predictions of sensory signals are fed back into the reservoir, alongside the prediction error, the discrepancy between the actual sensory signal and its prediction. If this learning process is successful, a well-trained reservoir minimizes prediction errors, thereby enabling the reservoir and the predictive layer to function as an autonomous dynamical system capable of ongoing sensory input generation. When discrepancies arise between predictions and actual sensory data, the reservoir's internal states are adjusted accordingly. This process of error-based correction plays a crucial role in the continuous generation of sensory input predictions within the predictive layer. This model is used as the basis for an integrated network model that integrates multiple sensory signals as depicted in Figure 1B.

To process multi-sensory information, the integrated network model is composed of lower areas, each dedicated to a specific sensory modality, and a higher area that integrates these lower areas. The state of each sensory reservoir in the lower areas is dimensionally reduced and temporally smoothed, creating a refined representation of sensory signals. The integration reservoir in the higher area processes these refined representations from the sensory reservoirs. The prediction generated by the integration reservoir is divided by sensory modality and sent as a top-down signal to the lower areas, where it is compared to the refined representation to compute the prediction error. This prediction error is then sent back to the integration reservoir as a bottom-up signal, prompting the integration reservoir to update its state in order to minimize the prediction error. Additionally, the prediction error is used to update the sensory reservoirs in the lower areas. The integration reservoir is trained to minimize the prediction error for the states of the lower areas, ultimately obtaining a unique representation that predicts multiple sensory inputs.

In the present study, we use leaky integrator (LI) neurons in the reservoir. LI neurons accumulate neural inputs in their internal state as membrane potential over time, and this membrane potential decays according to a specific time constant. The neurons' firing rate is determined by their membrane potential and is represented by a continuous value as the firing rate in rate-coding. In the reservoir-based predictive coding model of each area, the internal state *m*(*t*) changes according to the following equation:


(1) $$\begin{array}{l} m^{\left(i\right)}\left(t+\Delta t\right)=\left(1-\frac{\Delta t}{\tau^{\left(i\right)}}\right)m^{\left(i\right)}\left(t\right)+\frac{\Delta t}{\tau^{\left(i\right)}}I^{\left(i\right)}\left(t\right), \end{array}$$

where Δ*t* denotes the time step, and τ^(*i*)^ denotes the time constant of leaky-integration of the reservoir neurons. The superscript *i*∈{*V, A, I*} is used to represent the index of each area. The $r^{\left(i\right)}\left(t\right)\in {\mathbb{R}}^{N_{m}}$ denotes the firing rate of reservoir neurons. The firing rate of each neuron $r_{j}^{\left(i\right)}\left(t\right)$ is calculated with a non-linear activation function as $r_{j}^{\left(i\right)}\left(t\right)=\text{tanh}\left(m_{j}^{\left(i\right)}\left(t\right)\right)$, where the subscript *j* denotes the index of the neuron. The *I*^(*i*)^(*t*) denotes the neural input. The neural input to the local network that is modeled by the reservoir consisted of the input from the recurrent connection, feedback from the prediction, feedback from the prediction error, and the top-down signal from the higher area as described by the following equation:


(2) $$\begin{array}{l} I^{\left(i\right)}\left(t\right)=W_{\text{rec}}^{\left(i\right)}r^{\left(i\right)}\left(t\right)+W_{\text{back}}^{\left(i\right)}y^{\left(i\right)}\left(t\right)+W_{\text{err}}^{\left(i\right)}e^{\left(i\right)}\left(t\right)-b^{\left(i\right)}\left(t\right). \end{array}$$

The $W_{\text{rec}}^{\left(i\right)}\in {\mathbb{R}}^{N_{m}^{\left(i\right)}\times N_{m}^{\left(i\right)}}$, $W_{\text{back}}^{\left(i\right)}\in {\mathbb{R}}^{N_{m}^{\left(i\right)}\times N_{y}^{\left(i\right)}}$, and $W_{\text{err}}^{\left(i\right)}\in {\mathbb{R}}^{N_{m}^{\left(i\right)}\times N_{y}^{\left(i\right)}}$ denote the recurrent connection, the feedback connection of prediction $y^{\left(i\right)}\in {\mathbb{R}}^{N_{y}}$, and the feedback connection of prediction error $e^{\left(i\right)}\in {\mathbb{R}}^{N_{y}}$, respectively. These matrices are initially configured randomly with connection strengths $\alpha_{\text{rec}}^{\left(i\right)}$, $\alpha_{\text{back}}^{\left(i\right)}$, and $\alpha_{\text{err}}^{\left(i\right)}$, and the connectivity $\beta_{\text{rec}}^{\left(i\right)}$, $\beta_{\text{back}}^{\left(i\right)}$, and $\beta_{\text{err}}^{\left(i\right)}$, respectively; the matrices have $\beta_{\text{rec}}^{\left(i\right)}\times N_{m}^{\left(i\right)}\times N_{m}^{\left(i\right)}$, $\beta_{\text{back}}^{\left(i\right)}\times N_{m}^{\left(i\right)}\times N_{y}^{\left(i\right)}$, and $\beta_{\text{err}}^{\left(i\right)}\times N_{m}^{\left(i\right)}\times N_{y}^{\left(i\right)}$ non-zero elements, respectively. The non-zero elements are randomly chosen from sets $\left\{-\alpha_{\text{rec}}^{\left(i\right)},\alpha_{\text{rec}}^{\left(i\right)}\right\}$, $\left\{-\alpha_{\text{back}}^{\left(i\right)},\alpha_{\text{back}}^{\left(i\right)}\right\}$, and $\left\{-\alpha_{\text{err}}^{\left(i\right)},\alpha_{\text{err}}^{\left(i\right)}\right\}$ with equal probability, respectively. These matrices are fixed during experiments.

Each local network generates the prediction of the sensory signal or bottom-up signals from lower areas, based on the activity of the neurons. In the PCRC model, the prediction is represented as the linear combination of the firing rate by the following equation:


(3) $$\begin{array}{l} y^{\left(i\right)}\left(t\right)=W_{\text{out}}^{\left(i\right)}r^{\left(i\right)}\left(t\right), \end{array}$$

where *W*_out_ denotes the readout connection to generate prediction. The prediction error *e*^(*i*)^(*t*) is defined as the difference between the prediction *y*^(*i*)^(*t*) and the sensory input *d*^(*i*)^(*t*), expressed by *e*^(*i*)^(*t*) =*d*^(*i*)^(*t*)−*y*^(*i*)^(*t*).

The readout connection matrix $W_{\text{out}}^{\left(i\right)}$ is trained to minimize the prediction error. In this research, the readout connection matrix is updated based on the FORCE algorithm proposed by Sussillo and Abbott (2009), by using the local prediction error *e*^(*i*)^(*t*).


(4) $$\begin{array}{l} P^{\left(i\right)}\left(0\right)=\frac{E}{\alpha_{f}^{\left(i\right)}}, \end{array}$$

(5) $$\begin{array}{l} P^{\left(i\right)}\left(t\right)=P^{\left(i\right)}\left(t-\Delta t\right)-\frac{P^{\left(i\right)}\left(t-\Delta t\right)r^{\left(i\right)}\left(t\right){r^{\left(i\right)}}^{T}\left(t\right){P^{\left(i\right)}}^{T}\left(t-\Delta t\right)}{1+{r^{\left(i\right)}}^{T}\left(t\right)P^{\left(i\right)}\left(t-\Delta t\right)r^{\left(i\right)}\left(t\right)}, \end{array}$$

(6) $$\begin{array}{l} W_{\text{out}}^{\left(i\right)}\left(t+\Delta t\right)=W_{\text{out}}^{\left(i\right)}\left(t\right)+e^{\left(i\right)}\left(t\right){\left\{P^{\left(i\right)}\left(t\right)r^{\left(i\right)}\left(t\right)\right\}}^{T}, \end{array}$$

where the *P*^(*i*)^ denotes the inverse of the auto-correlation matrix of the firing rate *r*^(*i*)^(*t*), and $\alpha_{f}^{\left(i\right)}$ denotes the regularization parameter. The *E* denotes the identity matrix. The FORCE algorithm is applied to the model only during the training phase. Otherwise, the model obtains external information only through the prediction error signal.

The model introduces the modulation of the prediction error feedback to reproduce the multi-sensory integration by reliability-weighting. Since the integration reservoir receives bottom-up signals as the prediction error feedback, the weights for the bottom-up signal, representing the spoken word in each modality, can be formalized as follows:


(7) $$\begin{array}{l} e^{\left(I\right)}\left(t\right)=\left[\alpha_{e}^{\left(IA\right)}e^{\left(IA\right)}\left(t\right),\alpha_{e}^{\left(IV\right)}e^{\left(IV\right)}\left(t\right)\right], \end{array}$$

where $\alpha_{e}^{\left(IA\right)}$ and $\alpha_{e}^{\left(IV\right)}$ denote the strength of the prediction error feedback of auditory and visual bottom-up signals, respectively. The precision has been proposed to represent the reliability of the prediction as one of the components of predictive coding in a Bayesian fashion (Shipp, 2016). The precision modulates the gain of prediction error neurons, modulating the signals between hierarchies. In the proposed model, the modulation of the bottom-up signal of each lower layer is represented by modulating the strength of prediction error feedback $\alpha_{e}^{\left(IA\right)}$,$\alpha_{e}^{\left(IV\right)}$ of the integration reservoir. The terms $e^{\left(IA\right)}\left(t\right)\in {\mathbb{R}}^{N_{y}^{\left(IA\right)}}$ and $e^{\left(IV\right)}\left(t\right)\in {\mathbb{R}}^{N_{y}^{\left(IV\right)}}$ denote the prediction errors of the integration reservoir for auditory and visual modalities, calculated as follows:


(8) $$\begin{array}{l} {ẽ}_{j}^{\left(I\right)}=d_{j}^{\left(I\right)}-y_{j}^{\left(I\right)}\left(j\in \left\{1,2,\cdots \;,N_{y}^{\left(I\right)}\right\}\right), \end{array}$$

(9) $$\begin{array}{r} e^{\left(IA\right)}={\left({ẽ}_{1}^{\left(I\right)},{ẽ}_{2}^{\left(I\right)},\cdots \;,{ẽ}_{N_{y}^{\left(IA\right)}}\right)}^{T},\\ e^{\left(IV\right)}={\left({ẽ}_{N_{y}^{\left(IA\right)}+1}^{\left(I\right)},{ẽ}_{N_{y}^{\left(IA\right)}+2}^{\left(I\right)},\cdots \;,{ẽ}_{N_{y}^{\left(IA\right)}+N_{y}^{\left(IV\right)}}^{\left(I\right)}\right)}^{T}, \end{array}$$

where $d_{j}^{\left(I\right)}$ denotes the element of the bottom-up signal *d*^(*I*)^. The bottom-up signal *d*^(*I*)^(*t*) is calculated as follows:


(10) $$\begin{array}{l} {\tilde{d}}^{\left(I\right)}\left(t\right)=\left[U_{A}^{-1}r^{\left(A\right)}\left(t\right),U_{V}^{-1}r^{\left(V\right)}\left(t\right)\right], \end{array}$$

(11) $$\begin{array}{l} d^{\left(I\right)}\left(t+\Delta t\right)=d^{\left(I\right)}\left(t\right)+\frac{\Delta t}{\tau_{d}}\left({\tilde{d}}^{\left(I\right)}\left(t\right)-d^{\left(I\right)}\left(t\right)\right), \end{array}$$

where τ_*d*_ denotes the time constant of smoothing, and the $U_{A}^{-1}$ and $U_{V}^{-1}$ denote the dimension reduction matrices of the firing rate of the visual reservoir and auditory reservoir, respectively. The top-down signals for auditory and visual modalities, *b*^(*A*)^ and *b*^(*V*)^ are derived from the prediction error of the integration reservoir as follows:


(12) $$\begin{array}{l} b^{\left(A\right)}\left(t\right)=U_{A}e^{\left(IA\right)}\left(t\right),b^{\left(V\right)}\left(t\right)=U_{V}e^{\left(IV\right)}\left(t\right), \end{array}$$

with *U*_*A*_ and *U*_*V*_ representing the decomposition matrices for auditory and visual reservoir firing rates, respectively.

The proposed model is trained through the following steps. Initially, each lower area is individually trained to accurately predict the sensory signal from the training dataset. Subsequently, the state-collecting matrices are constructed from the time series of the state of the lower area reservoirs, which are driven by the training data. Next, the decomposition matrices, *U*_*A*_ and *U*_*V*_, are derived from these state-collecting matrices through principal component analysis. Concurrently, the dimension reduction matrices, ${U_{A}}^{-1}$ and ${U_{V}}^{-1}$, are determined as the pseudo-inverse of *U*_*A*_ and *U*_*V*_, respectively. Finally, the entire model is trained using the training data by integrating the outputs from each area.

The dataset used for training the model is CUAVE, an audiovisual speech perception dataset (Patterson et al., 2002). This dataset offers speech sequences as auditory signals and the corresponding faces of speakers as video footage. For our experiments, data from five speakers were selected and split into training and validation datasets at a ratio of 3:2. The auditory signals were processed using Lyon's cochlear filter (Lyon, 1982), producing a cochleagram for each spoken word. The visual data, specifically images of the speaker's face, were preprocessed using the method proposed by Ngiam et al. (2011). This process involved extracting the 32 principal components of the images around the speakers' mouth and their time derivatives for each spoken word. To standardize the data, each preprocessed spoken word was adjusted to have the same time step (8 ms) and duration (1.6 s).

**Figure 2:**
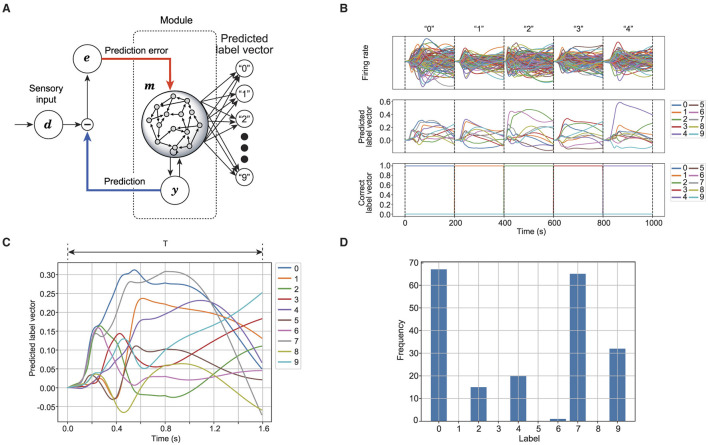
Schematic of speech recognition in reservoir computing. **(A)** Shows a readout connection to generate a predicted label vector from the reservoir. **(B)** Shows the time series of the reservoir while outputting the predicted label vector. The first row shows the firing rate of the auditory reservoir. The second row shows the time series of the predicted label vector. The third row shows the time series of the correct label vector. **(C)** Shows the time series of predicted label vector for a spoken word. **(D)** Shows the frequency for each label that is predicted from the time series shown in **(C)**.

The proposed model's performance in speech recognition is evaluated to verify whether the reservoir neurons correctly represent the spoken words. For speech recognition, the model extracts a vector representing the predicted label from the reservoir (Verstraeten et al., 2005). Figure 2 shows the process of label prediction using the reservoir. For each time step, the label vector $l^{\left(i\right)}\left(t\right)\in {\mathbb{R}}^{\left|W\right|}$ is derived from the firing rate of reservoir neurons as follows:


(13) $$\begin{array}{l} l^{\left(i\right)}\left(t\right)=W_{\text{label}}^{\left(i\right)}r^{\left(i\right)}\left(t\right), \end{array}$$

where $W_{\text{label}}^{\left(i\right)}\in {\mathbb{R}}^{|W|\times N_{m}^{\left(i\right)}}$ is the readout matrix obtained by ridge regression for the training dataset. W represents a set of spoken words, including 10 different digits: W={0,1,⋯,9} and the number of spoken words |W|=10. The correct label vector ${\hat{l}}^{\left(i\right)}$ uses one-hot encoding, where one element, corresponding to the index of the spoken word, is set to 1.0, and the rest are set to 0, as depicted in the third row of Figure 2B. The predicted label *L*^(*i*)^(*t*) is determined from the label vector using $L^{\left(i\right)}\left(t\right)=\underset{w\in W}{\mathrm{argmax}}\;l_{w}^{\left(i\right)}\left(t\right)$. As Figure 2 shows, the reservoir responds to the given sensory signal, translating the activity into the predicted label vector, as shown in Figure 2C. The most frequently predicted label $L=\text{MODE}\left({\left\{L^{\left(i\right)}\left(n\cdot \Delta t\right)\right\}}_{n=0}^{\frac{T}{\Delta t}-1}\right)$ is assigned as the predicted word. Figure 2D shows the frequency of each label predicted for the given word over *T* seconds. Accuracy is defined as the proportion of words correctly predicted for the validation dataset.

To evaluate the contribution of reservoir computing to speech recognition, the temporal recognition accuracy is analyzed. Temporal recognition accuracy is defined by $S^{\left(i\right)}\left(T\right)=\frac{n\left(T\right)}{\left|D\right|}$, where *n*(*T*) denotes the frequency that the label is predicted correctly while *T* seconds time series is used for recognition. D represents the validation dataset. The temporal recognition accuracy *S*^(*i*)^(*T*) is assumed to be larger when the time width of label prediction is increased because the model uses the sensory information over multiple time steps. If the temporal recognition accuracy decreases beyond a certain time width of label prediction, it is assumed that essential information has been lost in the past.

In the experiments, the following parameters are used: $N_{m}^{\left(A\right)}=500$, $N_{m}^{\left(V\right)}=500$, $N_{m}^{\left(I\right)}=500$, $N_{y}^{\left(A\right)}=86$, $N_{y}^{\left(V\right)}=64$, $N_{y}^{\left(IA\right)}=20$, $N_{y}^{\left(IV\right)}=20$, τ^(*A*)^ = 270(ms), τ^(*V*)^ = 380(ms), τ^(*I*)^ = 300(ms), $\alpha_{\text{rec}}^{\left(A\right)}=0.99$, $\alpha_{\text{rec}}^{\left(V\right)}=0.99$, $\alpha_{\text{rec}}^{\left(I\right)}=0.99$, $\alpha_{\text{err}}^{\left(A\right)}=0.1$, $\alpha_{\text{err}}^{\left(V\right)}=0.1$, $\alpha_{\text{err}}^{\left(I\right)}=0.1$, $\alpha_{\text{back}}^{\left(A\right)}=0.1$, $\alpha_{\text{back}}^{\left(V\right)}=0.1$, $\alpha_{\text{back}}^{\left(I\right)}=0.1$, $\beta_{\text{rec}}^{\left(A\right)}=0.1$, $\beta_{\text{rec}}^{\left(V\right)}=0.1$, $\beta_{\text{rec}}^{\left(I\right)}=0.1$, $\beta_{\text{back}}^{\left(A\right)}=0.1$, $\beta_{\text{back}}^{\left(V\right)}=0.1$, $\beta_{\text{back}}^{\left(I\right)}=0.1$, $\beta_{\text{err}}^{\left(A\right)}=0.1$, $\beta_{\text{err}}^{\left(V\right)}=0.1$, $\beta_{\text{err}}^{\left(I\right)}=0.1$, τ_*d*_ = 80(ms), $\alpha_{f}^{\left(A\right)}=1.0$, $\alpha_{f}^{\left(V\right)}=1.0$, $\alpha_{f}^{\left(I\right)}=1.0$.

## 3 Results

**Figure 3:**
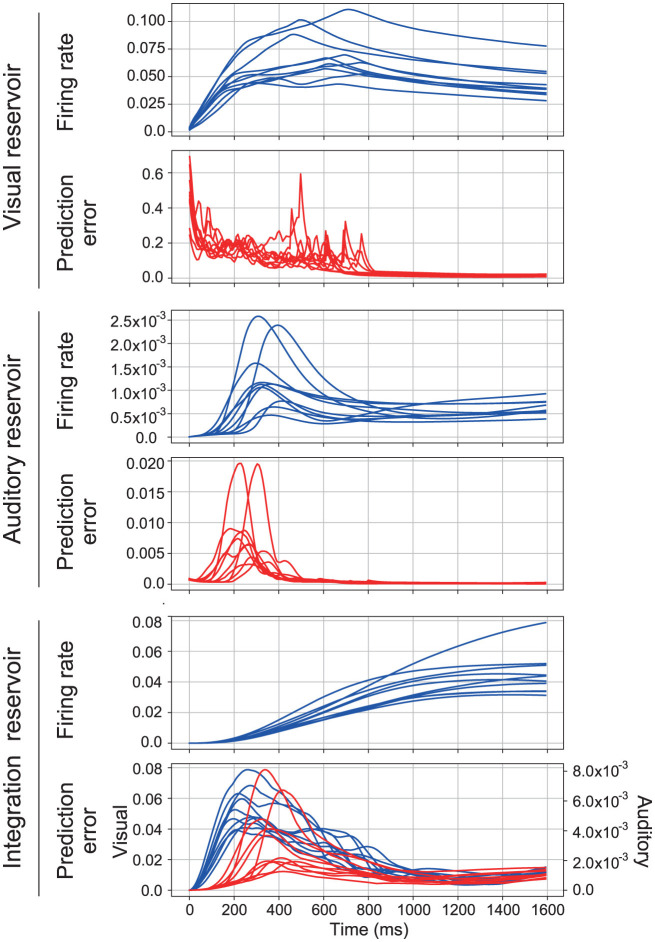
Typical time series of the proposed model. It consists of three sections, each corresponding to a different reservoir. In each section, the first row shows the firing rate and the second row shows the prediction error. The first section shows the time series of the visual reservoir. The second section shows the time series of the auditory reservoir. The third section shows the time series of the integration reservoir. In the third section, the prediction error of visual (blue lines) and auditory signals (red lines) is represented separately.

In the proposed model, the reservoir in each area is driven by the prediction error associated with sensory signals, whereas the reservoir in the higher integration area is driven by the prediction error related to the activity from lower sensory areas. A typical response of the proposed model for 10 samples of the spoken word “1” is shown in Figure 3. Within this figure, the firing rate is defined as ${\bar{r}}^{\left(i\right)}\left(t\right)=\overset{N_{m}^{\left(i\right)}}{\underset{j=1}{\sum }}|r_{j}^{\left(i\right)}\left(t\right)|/N_{m}^{\left(i\right)}$, and the prediction error is quantified as ${ē}^{\left(i\right)}\left(t\right)=\overset{N_{y}^{\left(i\right)}}{\underset{j=1}{\sum }}|e_{j}^{\left(i\right)}\left(t\right)|/N_{y}^{\left(i\right)}$. Note that the prediction error of the integration reservoir is displayed for each sensory modality, separately. This separation facilitates a direct comparison between the different modalities. Typically, within each reservoir, an initial increase in the prediction error for the presented sensory signal is observed, followed by a subsequent rise in the firing rate and then a decrease in the prediction error. This sequence of events underscores the model's dynamical response to varying inputs and indicates its capacity to adapt and process sensory information effectively.

**Figure 4:**
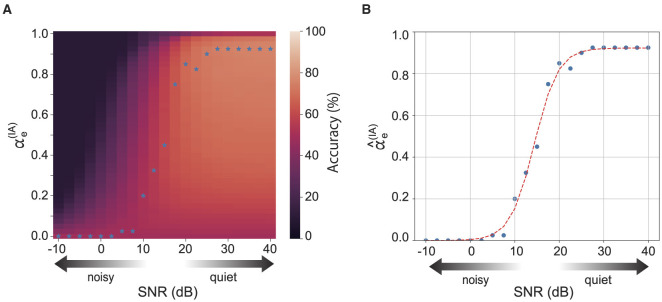
**(A)** Shows the accuracy of the integration reservoir for various levels of auditory noise and the strength of auditory prediction error feedback. The blue stars represent the optimal strength of auditory prediction error feedback ($\alpha_{e}^{\left(IA\right)}$) that yields the highest accuracy for each noise level. **(B)** Shows the optimal $\alpha_{e}^{\left(IA\right)}$ for each noise strength. The red dashed line represents the fitting curve.

The recognition accuracy of the integration reservoir depends on the strength of the feedback, i.e., how much sensory information is incorporated into the reservoir (Figures 1A, B). Figure 4 displays the recognition accuracy for each reservoir for the levels of auditory noise and the auditory prediction error feedback $\alpha_{e}^{\left(IA\right)}$. Note that the total strength of prediction error feedback is maintained at a constant sum, with $\alpha_{e}^{\left(IA\right)}+\alpha_{e}^{\left(IV\right)}=1$ in order to confirm the effect of the balance of sensory information. The optimal feedback strength for the best recognition accuracy (blue stars shown in Figure 4) depends on the noise intensity and the strength of auditory prediction error feedback (Figure 4A). We confirmed the relational equation between the optimal strength of auditory prediction error and the noise intensity. Figure 4B shows the optimal strength of the auditory prediction error feedback for each auditory noise level. The blue dots represent the optimal strength of the auditory prediction feedback obtained by the above optimization process. The relationship between the optimal feedback strength and the noise can be fitted with a sigmoidal shape inference model, which is based on conventional multi-sensory integration theory:


(14) $$\begin{array}{l} \alpha_{e}^{\left(IA\right)}=\frac{\alpha_{\text{max}}}{1+\exp\left(-a\left(x-x_{0}\right)\right)}, \end{array}$$

where *x* denotes the level of auditory noise, and α_max_, *a*, and *x*_0_ are model parameters. The red dashed line illustrates the model's fit to the experimental data. According to Figure 4B, the inference model fits well to the experimental result.

**Figure 5:**
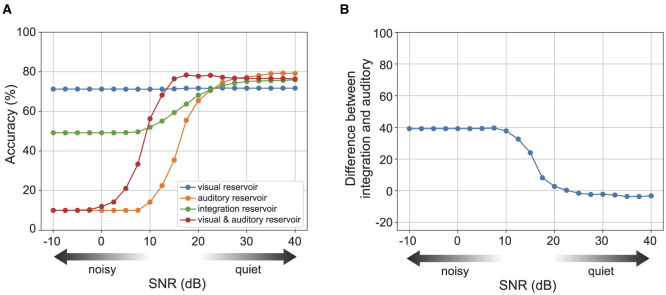
The recognition accuracy of each reservoir for the strength of auditory noise. Here, we set $N_{m}^{\left(I\right)}=500$. **(A)** Shows the accuracy of each reservoir for the noise strength. **(B)** Shows the difference in accuracy between the integration reservoir and the auditory reservoir. See Supplementary Figure S1 for the $N_{m}^{\left(I\right)}=1,000$ case.

Figure 5A displays the recognition accuracy for each reservoir across varying levels of auditory noise. The blue curve shows the accuracy of the visual reservoir, and the orange curve shows the accuracy of the auditory reservoir. While the visual reservoir's recognition accuracy exhibits minimal changes, the auditory reservoir's accuracy diminishes significantly in the presence of high auditory noise levels. The green dots and line show the accuracy of the integration reservoir with the optimal strength of auditory prediction error feedback for each noise level. The integration reservoir's recognition accuracy remains robust and less affected, even with increased auditory noise strength.

We also confirmed the contribution of the integration reservoir to perform robust recognition. The red curve shows the accuracy of concatenated sensory reservoir state [*r*^(*A*)^, *r*^(*V*)^]. The performance of the concatenated reservoir keeps higher accuracy than the integration reservoir under relatively lower auditory noise levels. Conversely, for relatively higher auditory noise levels, the performance of the concatenated reservoir diminishes significantly.

We investigated the effect of modulating the strength of auditory prediction error feedback. The superiority of utilizing multi-sensory information over uni-modal sensory information, namely, an increase in recognition accuracy in noisy environments (van de Rijt et al., 2019). Figure 5B shows the difference of accuracy between the integration reservoir with the modulation of the strength of auditory prediction error feedback and the auditory reservoir. The superiority increases for the levels of auditory noise.

**Figure 6:**
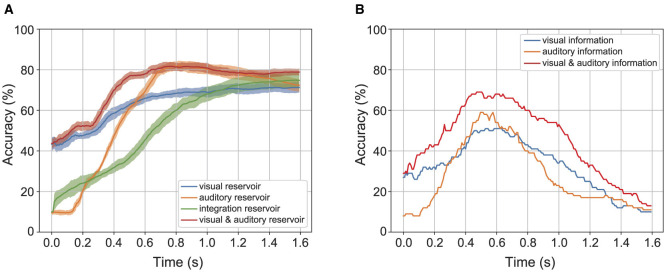
Temporal accuracy of the reservoir and the direct readout from the sensory information. **(A)** Shows the accuracy of the reservoir. **(B)** Shows the accuracy of direct readout from the sensory information.

Reservoirs achieve pattern recognition by temporarily storing time-series information from sensor signals. Their contribution is assessed by temporal recognition accuracy, i.e., the estimated value of the labels obtained from a series of state vectors in a certain time window. Here, the recognition accuracy obtained from the state vector of the reservoir is compared with the recognition accuracy obtained from the state vector of the preceding sensor signal. Figure 6 shows the temporal recognition accuracy for the time width of label prediction under 40dB auditory noise. Figure 6A shows the temporal recognition accuracy of the label predicted from the reservoirs. The color band represents the standard deviation of temporal recognition accuracy, with its width set to 2σ for the standard deviation σ. Figure 6B represents the temporal recognition accuracy of the label directly predicted from the sensory information using ridge regression. According to Figure 6A, the temporal recognition accuracy saturate with the time width 0.6(s) when the label is predicted from the reservoir. According to Figure 6B, the temporal recognition accuracy gets the peak with time width 0.6(s) and decreases above the time width. These results show that the recognition accuracy obtained from the reservoir is higher than that of the preceding sensor stage.

The proposed model maintains optimal recognition in situations where auditory noise fluctuates over time by estimating the noise intensity and dynamically adapting the feedback strength. The noise intensity is estimated as the moving average of auditory prediction error ${ē}_{\text{avg}}^{\left(A\right)}\left(t\right)$ as follows:


(15) $$\begin{array}{l} {ē}_{\text{avg}}^{\left(A\right)}\left(t\right)=\left(1-\frac{\Delta t}{\tau_{\text{avg}}}\right){ē}_{\text{avg}}^{\left(A\right)}\left(t\right)+\frac{\Delta t}{\tau_{\text{avg}}}\overset{N_{y}^{\left(A\right)}}{\underset{i=1}{\sum }}\frac{{e_{i}}^{2}\left(t\right)}{N_{y}^{\left(A\right)}}. \end{array}$$

The optimal feedback strength $\alpha_{e}^{\left(IA\right)}\left(t\right)$ is computed as Equation 14 with the estimated level of auditory noise *x*′ as follows:


(16) $$\begin{array}{l} x^{\prime }\left(t\right)=c\sqrt{{ē}_{\text{avg}}^{\left(A\right)}\left(t\right)}+b, \end{array}$$

(17) $$\begin{array}{l} \alpha_{e}^{\left(IA\right)}\left(t\right)=\frac{\alpha_{\text{max}}}{1+\exp\left(-a\left(x^{\prime }\left(t\right)-x_{0}\right)\right)}. \end{array}$$

The parameters are set as follows: *c* = −441, *b* = 9.44, τ_avg_ = 3.2(s), α_max_ = 0.92, *x*_0_ = 14, and *a* = 0.37. The coefficient *c* and the bias *b* are estimated experimentally in advance.

**Figure 7:**
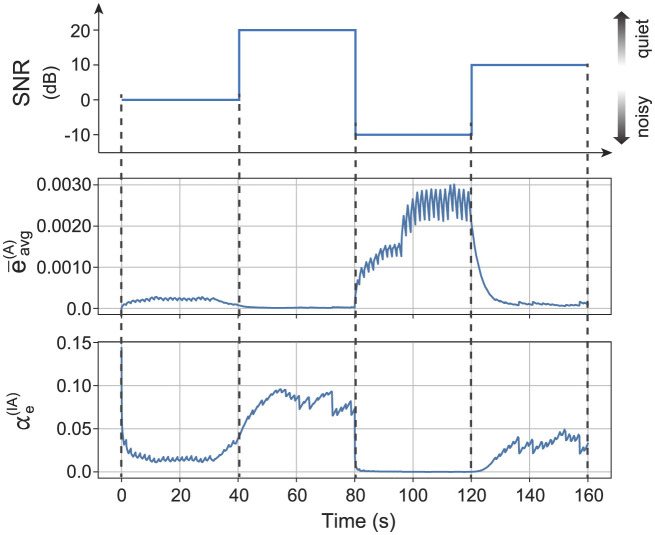
Typical time series while dynamic modulation of prediction error feedback of the integration reservoir based on the experimental obtained relationship between the noise strength and the optimal strength of prediction error feedback. The first row represents the auditory noise strength. The second row shows the moving average of the auditory prediction error ${ē}_{\text{avg}}^{\left(A\right)}$ of auditory reservoir. The third row shows the strength of auditory prediction error feedback $\alpha_{e}^{\left(IA\right)}$ of the integration reservoir.

Dynamic adjustment of the intensity of the prediction error feedback realizes proper perception even when the noise intensity fluctuates. Figure 7 shows the typical time series of dynamic modulation of the prediction error feedback in the integration reservoir. The auditory noise strength of the sensory input changes over time. The moving average of the prediction error in the auditory reservoir increases as the auditory noise strength increases. The strength of auditory prediction error feedback in the integration reservoir adjusts based on the moving average of the prediction error in the auditory reservoir. Specifically, the auditory prediction error feedback strength is suppressed when the auditory noise level is relatively high (0 dB to –10 dB). In contrast, the feedback strength is enhanced when the auditory noise level is relatively low (20 dB to 10 dB). We also confirmed that the integration reservoir achieves a mean recognition accuracy of 49.9(%) with the dynamic modulation of $\alpha_{e}^{\left(IA\right)}\left(t\right)$.

## 4 Discussion

In this research, we proposed a multi-sensory integration model based on the idea of predictive coding, reservoir computing, and reliability weighting. This model demonstrates robust recognition capabilities in the presence of sensory noise on the multi-modal speech recognition task.

In this model, the dynamics within a network with recurrent connections are crucial for multi-sensory information processing. Auditory and visual reservoirs are trained to reconstruct the sensory signals of each modality, acting as short-term temporal storage for the sensory signals. Furthermore, the states of these recurrent networks are transmitted to a higher-level integration network in the integration area. The network within the integration area is responsible for reconstructing the unified representation of the two modalities and maintaining the integrated information. Based on this integrated information, pattern recognition is performed through a linear regression of the reservoir's state in the integration area, which outputs the appropriate label. This process facilitates the transfer of information from the two sensory areas to the integration area via feedback on prediction errors. The reliability weighting mechanism adjusts the strength of this feedback based on the reliability of the signals, achieving a robust system capable of operating in environments with noisy sensory signals.

While this mechanism works to perform robust recognition, in low SNR situations, the recognition accuracy of the integration reservoir is lower than that of the visual reservoir. Although the integration reservoir receives both auditory and visual signals during training, it does not receive the auditory signal under noisy conditions, which prevents it from adapting to the unknown signal. This tendency can be interpreted as being caused by overfitting in the integration reservoir. Ideally, the integration reservoir should adapt to the signal when sensory noise is strong and achieve the highest accuracy.

A cognitive study by van de Rijt et al. (2019) reported a similar phenomenon, where recognition performance with audiovisual information was lower than in the visual-only condition, which is consistent with our model. van de Rijt et al. (2019) attributed this to an attention mechanism that divides different streams of sensory information. The results of our model suggest that overfitting in the integration area to bottom-up signals contributes to the degraded recognition, as the degradation occurs when $\alpha_{e}^{\left(IA\right)}=0$ (Figure 5A).

Analysis of the temporal recognition accuracy between the estimated labels and the correct labels, based on a time frame for the activity pattern of the reservoir, indicates that the reservoir enhances recognition accuracy by providing short-term memory of the temporal structure of speech. The temporal recognition accuracy reflects how the reservoir correctly represents a given spoken word correctly for the time width used to determine the predicted label. As shown in Figure 6, the temporal recognition accuracy of the model increases from the moment the sensory signal is given. In contrast, the temporal recognition accuracy decreases after peaking when the label vector is readout from sensory signals directly, suggesting that the sequence after the peak lacks useful information for speech recognition. These properties indicate that the dynamics occurring within the recurrent network contribute to speech recognition.

The principle of inverse effectiveness is a key feature of multi-sensory integration, indicating that the enhancement of combining multiple sensory inputs increases as the sensory signal decreases. In tasks involving multi-sensory speech perception, it is observed that the enhancement in recognition accuracy from multi-sensory stimuli becomes more significant with higher levels of auditory noise (Stevenson and James, 2009). Consistently, our model demonstrates that the improvement in recognition accuracy within the integration reservoir is more pronounced under conditions of increased auditory noise, as illustrated in Figure 5B. This result is consistent with experimental findings (van de Rijt et al., 2019). For a comparison of the relationship between perception accuracy and auditory noise, see Figures 4, 5 in this article and Figures 6, 7 in the study by van de Rijt et al. (2019).

A neural implementation of predictive coding theory is organized by Shipp (2016). For a comparison of the relationship between the proposed model and the conventional predictive coding architecture, see Figure 1B in this article and Figures 1, 3 in the study by Shipp (2016). In our model, the internal connections in each layer of predictive coding theory are represented by randomly connected neurons, or reservoirs. The prediction error between the state of the lower area and the prediction from the higher area is represented in the lower area and sent to the higher area as a bottom-up signal. The prediction of the state of the lower area is represented in the higher area, and the prediction signal is sent to the lower area. This arrangement is consistent with the neural implementation of predictive coding as organized by Shipp (2016). The reliability weighting mechanism in our model can be interpreted as the precision mechanism that modulates neuronal signals in predictive coding theory. This mechanism is implemented by weighting the projection of prediction errors to the integration reservoir using the parameters $\alpha_{e}^{\left(IA\right)}$ and $\alpha_{e}^{\left(IV\right)}$.

The specific brain structures involved in multi-sensory speech recognition have been investigated in previous studies (Sekiyama et al., 2003, Nath and Beauchamp, 2011). The mechanism of weighting sensory information is supported by the functional connectivity among cortical regions. For example, Nath and Beauchamp (2011) reported variability in the functional connectivity between the visual cortex, auditory cortex, and superior temporal sulcus (STS). In our model, the visual cortex and auditory cortex correspond to the visual and auditory reservoirs, respectively, while the multi-sensory area (STS) corresponds to the integration area. The strength of the neural signals among areas in our model is represented by the strength of prediction error feedback. The experimental results of our model suggest that the modulation of prediction error signal strength may underlie the modulation of functional connectivity during multi-sensory speech recognition.

The computational model for multi-sensory speech recognition, as proposed by Ma et al. (2009), extends beyond the traditional multi-sensory integration models based on Bayesian inference, particularly for multi-class tasks. Yet, the specific neural mechanisms that facilitate the integration of visual and auditory signals-each with its own unique set of dimensions and temporal variations-remain to be fully understood. Our model seeks to overcome this limitation by implementing a hierarchical structure that includes a randomly configured recurrent network, thereby showcasing the capability of recurrent structures for managing the multi-sensory integration process. As mentioned by Enel et al. (2016), the recurrent structure of the local network of the cortex has the rich properties to represent time contextual information of the sensory signals. Our results indicate that the local connectivity traits of the cortex significantly contribute to enhancing multi-sensory integration.

We demonstrated the specific methods for adjusting the intensity of reliability weighting. The method involves optimizing recognition accuracy by adjusting the weights based on the noise intensity for each sensory modality. As illustrated in Figure 4B, the relationship between the optimal feedback intensity for accurate recognition and the magnitude of noise in the sensory signals follows a sigmoid curve. This feedback mechanism can be established based on the noise intensity estimated in each sensory modality's region as shown in Figure 7. Moreover, the reliability weighting across different sensory modalities is linked to attentional mechanisms. Certain tasks may be efficiently accomplished by directing attention, either consciously or unconsciously, toward a specific sensory modality.

Other future work includes the analysis of more physiologically sophisticated neural models. In the present model, we used a firing rate model, but it will be necessary to develop a network model based on spiking neurons. Additionally, the plasticity of local connections within the recurrent network structure can be explored. A possible extension is the incorporation of a learning rule that leverages internal dynamics, rather than relying on a randomly connected network. It is also important to investigate how typical neural structures, such as receptive fields, are realized within the recurrent network structure under the framework of predictive coding theory. Various formulations of hierarchical structures could be explored as well. Deepening the network is a potential reformulation that could provide insights into the benefits of hierarchical structures in sensory processing streams, such as the primary and secondary visual cortices. As shown in Figure 4A, the integration area primarily relies on visual information under noisy conditions, suggesting that adding structures specific to each modality could improve model performance. In the context of reservoir computing, deepening the network is a common approach. For example, in our previous research (Yonemura and Katori, 2021), we demonstrated a hierarchical predictive coding model using reservoir computing. Moving forward, it will be necessary to analyze in detail how network topology and the properties and parameters of associated synapses contribute to multi-modal integration.

In summary, the proposed model not only replicates the characteristics of multi-sensory integration in a speech perception task but also provides insights into the neural basis underlying this integration. It highlights how the random recurrent structure plays a crucial role in representing the features of multi-sensory time series, aligning with conventional computational theories. This suggests that multi-sensory integration leverages a common neural framework in the cortex, facilitated by random recurrent connections. Finally, the findings from this research contribute to a deeper understanding of the cortical structure's role in the multi-sensory speech perception process.

## Data Availability

The original contributions presented in the study are included in the article/Supplementary material, further inquiries can be directed to the corresponding author.
