# Supplementary material for: Dynamical predictive coding with reservoir computing performs noise-robust multi-sensory speech recognition
Source: Front Comput Neurosci. 2024 Sep 23;18:1464603. doi: 10.3389/fncom.2024.1464603 (PMC11456454; doi:10.3389/fncom.2024.1464603)
Supplement: Supplementary file 1 [file Data_Sheet_1.PDF]

Supplementary Material

1 EFFECT OF NEURON SIZE ON RECOGNITION ACCURACY

Here, we analyze the effect of the number of neurons used to read out word labels. As shown in Figure 5(A), under quiet conditions (from 25 dB to 40 dB), the recognition accuracy of the concatenated reservoir with 1000 neurons is higher than that of the integration reservoir with 500 neurons. However, when the neuron count in the integration reservoir is increased to 1000, its accuracy surpasses that of the other reservoirs under quiet conditions, as shown in Figure S1(A). This result suggests that the lower accuracy observed in the integration reservoir with 500 neurons was due to the difference in neuron count compared to the concatenated reservoir. Additionally, Figure S1(B) demonstrates that the difference in accuracy between the integration reservoir and the auditory reservoir increases with higher levels of auditory noise, which is consistent with the results obtained when $N_m^{(I)} = 500$.

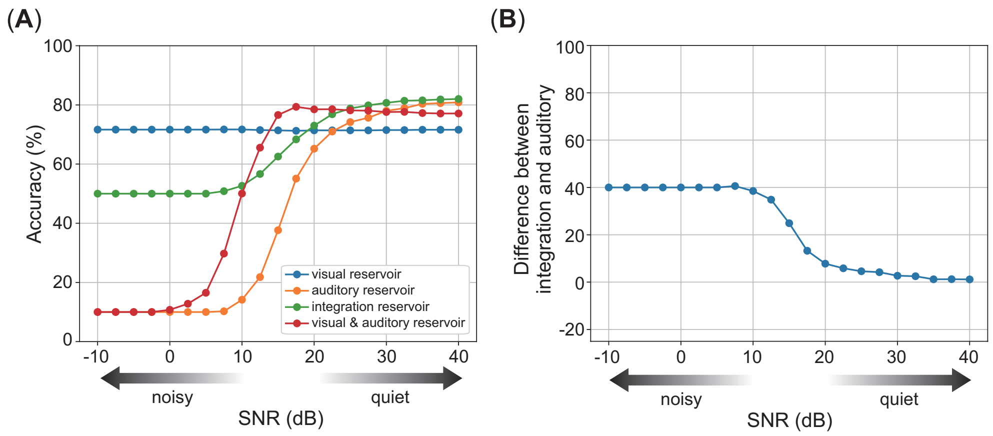

Figure S1. The recognition accuracy of each reservoir for the strength of auditory noise when $N_m^{(I)} = 1000$. **(A)** shows the accuracy of each reservoir for the noise strength. **(B)** shows the difference in accuracy between the integration reservoir and the auditory reservoir.

2 CONTRIBUTION OF PREDICTION ERROR ON RECOGNITION ACCURACY

We confirmed whether the prediction error unit contributes the recognition accuracy. We reformulated eq.(2), which represents the neural input of the reservoir, as follows:

$$\boldsymbol{I}^{(i)}(t) = W_{\mathrm{rec}}^{(i)}\boldsymbol{r}^{(i)}(t) + W_{\mathrm{back}}^{(i)}\boldsymbol{d}^{(i)}(t). \quad \text{(S1)}$$

To compare with the proposed model, the signal $\boldsymbol{d}^{(I)}(t)$ is replaced by $[\alpha_e^{(IA)}\boldsymbol{d}^{(IA)}(t), \alpha_e^{(IV)}\boldsymbol{d}^{(IV)}(t)]$, where $\boldsymbol{d}(t) = [\boldsymbol{d}^{(IA)}(t), \boldsymbol{d}^{(IV)}(t)]$. Figure S2 shows the recognition accuracy of the reformulated model, using the same parameters as in the experimental setting of the proposed model. As shown in Figure S2(A) and Figure 5(A), in quiet conditions, the accuracy of the reformulated model is higher than that of the proposed model. However, in noisy conditions, the accuracy of the reformulated model is lower than that of the proposed model. Additionally, Figure S2(B) demonstrates that the difference in accuracy

between the integration reservoir and the auditory reservoir increases with higher levels of auditory noise, which is consistent with the results observed in the model with prediction error.

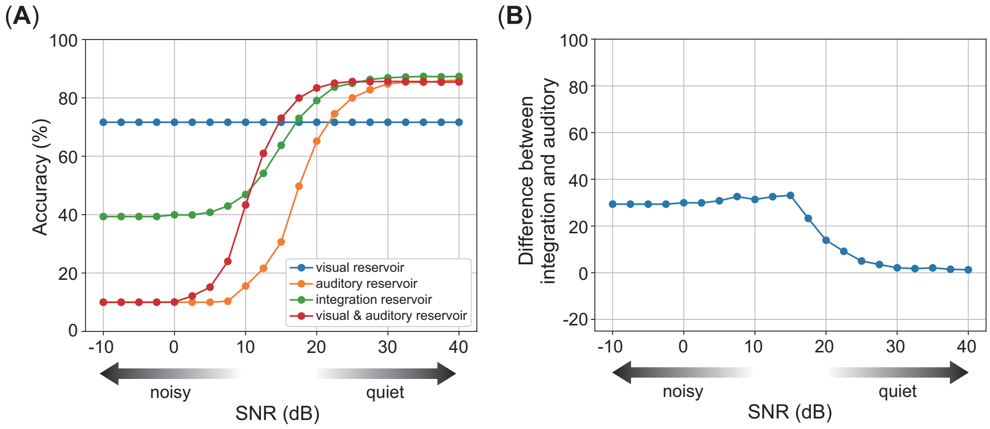

Figure S2. The recognition accuracy of the model without prediction error unit. **(A)** shows the accuracy of each reservoir for the noise strength. **(B)** shows the difference in accuracy between the integration reservoir and the auditory reservoir.
